# Physicochemical and Biological Examination of Two Glatiramer Acetate Products

**DOI:** 10.3390/biomedicines7030049

**Published:** 2019-07-03

**Authors:** Arthur Komlosh, Vera Weinstein, Pippa Loupe, Tal Hasson, Bracha Timan, Attila Konya, Jessica Alexander, Sigal Melamed-Gal, Steffen Nock

**Affiliations:** Specialty Research and Development, Teva Pharmaceutical Industries Ltd, Netanya 4250419, Israel

**Keywords:** glatiramer acetate, multiple sclerosis, physicochemical assays, non-biological complex drugs, Copaxone

## Abstract

Herein we compared 40 mg/mL lots of the active ingredient, glatiramer acetate, manufactured by Mylan/Natco to the active ingredient, glatiramer acetate in Copaxone (Teva Pharmaceuticals, Ltd., Netanya Israel) using physicochemical (PCC) methods and biological assays. No differences were seen between the Mylan/Natco and Teva lots with some low resolution release PCC assays (amino acid analysis, molecular weight distribution, interaction with Coomassie Brilliant Blue G-250). Changes in polydispersity between Mylan/Natco and Copaxone lots were found using size exclusion chromatography and the high resolution PCC method, known as Viscotek, and suggestive of a disparity in the homogeneity of mixture, with a shift towards high molecular weight polypeptides. Using RPLC-2D MALLS, 5 out of 8 Mylan/Natco lots fell outside the Copaxone range, containing a high molecular weight and high hydrophobicity subpopulation of polypeptides not found in Copaxone lots. Cation exchange chromatography showed differences in the surface charge distribution between the Copaxone and Mylan/Natco lots. The Mylan/Natco lots were found to be within Copaxone specifications for the EAE model, monoclonal and polyclonal binding assays and the in vitro cytotoxicity assay, however higher IL-2 secretion was shown for three Mylan/Natco lots in a potency assay. These observations provide data to inform the ongoing scientific discussion about the comparability of glatiramer acetate in Copaxone and follow-on products.

## 1. Introduction

Copaxone (glatiramer acetate, Teva Pharmaceuticals), a safe and effective treatment option for multiple sclerosis (MS), was approved by the United States Food and Drug Administration (FDA) in 1997 as a once daily 20 mg/mL injection, and in 2014 as a 40 mg/mL thrice weekly injection. MS is a neurodegenerative disease mediated by highly reactive lymphocytes which cross the blood brain barrier into the central nervous system (CNS), and cause widespread inflammation resulting demyelination, cell apoptosis and axonal loss [1]. The active ingredient of Copaxone is glatiramer acetate (GA), a non-biological complex drug (NBCD) composed of a heterogeneous mixture of nano-sized polypeptide components derived from the copolymerization of four synthetic amino acids (l-glutamic acid, l-alanine, l-tyrosine, and l-lysine), and was discovered to mimic myelin basic protein (MBP), an autoantigen involved in MS pathology as an altered peptide of MBP p82-100 [2,3]. The therapeutic effects of Copaxone are thought to be related to the presentation of Copaxone’s yet unidentified epitopes as antigens binding to various major histocompatibility complex Class II molecules on antigen-presenting cells and T-cells [3]. This interaction of the “immunological triad” leads to the generation of GA-specific T cells shifted from an MS-instigating pro-inflammatory to an anti-inflammatory T helper type 2 (Th2) phenotype [4,5], for a complete description, see Arnon and Aharoni 2019 [6].

Glatiramer acetate is more complex than a polypeptide or protein derived from a biotechnological process. The heterogeneous mixture of up to 10^29^ possible immunogenic polypeptides of varying sequences and sizes are impossible to isolate, quantify and sequence, or fully characterize, even with highly discriminatory analytical methods [7]. Therefore, while the composition of GA is defined by the specifics of multiple stages in the manufacturing process, the consistent quality of its active ingredient relies on the robustness of these manufacturing stages.

In 2017, Mylan received approval for 20 mg/mL and 40 mg/mL doses of their generic version of glatiramer acetate from the FDA [8]. The approval of the Abbreviated New Drug Application (ANDA) relied on physicochemical characterization and biological data to establish the sameness of the active ingredient, in accordance to the FDA guidance for industry for generic versions of GA, which does not necessitate clinical data [9]. In the European Union (EU), regulators decided on a different approach for follow-on GA (FOGA) products, relying not only on physicochemical and biological data, but also on clinical data to establish this similarity of the active substances [10]. In accordance, the EU FOGA is considered a hybrid product and not a generic version.

Since 2010, Teva Pharmaceuticals, the manufacturer of innovative Copaxone, has evaluated the physicochemical and biological characterizations of several FOGA products from various manufacturers using a battery of low and high resolution methods and functional assays, and has published the results of their analyses [11,12,13,14,15,16]. Collectively, the results of these examinations have shown the challenges associated with manufacturing a highly complex polypeptide mixture such as GA, and demonstrated the differences in the physicochemical and biological characteristics of the FOGA lots produced by multiple manufacturers. The present report describes Teva’s analyses of Mylan’s 40 mg/mL FOGA product lots, employing some of the quality control methods used for Copaxone release, along with high resolution sensitive methods and biological assays.

## 2. Materials and Methods

The tests were applied on eight lots of Mylan’s 40 mg/mL follow-on GA (FOGA) product (Lot numbers 170403, 170416, 170530, 170602, 170605, 170607, 170608, 170611 Mylan Pharmaceuticals Inc. Morgantown, WV, USA). For some of the tests (as specified), the Mylan 40 mg/mL lots were compared to Copaxone release specifications, and for other assays, the Mylan 40 mg/mL lots were compared to eight randomly chosen lots of Teva’s Copaxone 40 mg/mL (lot numbers C42306, C42331, C42335, C42336, C42338, C42339, C42341, C42342).

### 2.1. Low Resolution Release Tests

#### 2.1.1. Amino Acid Analysis (Relative Amino Acid Content)

Mylan FOGA samples were hydrolyzed in a 12% HCl solution, then incubated for 24 h at 110 °C. The hydrolysate was analyzed by high-pressure liquid chromatography-mass spectrometry (HPLC-MS), using a Primesep 100, 5 μm, 4.6 mm × 150 mm, column (SIELC, Wheeling, IL, USA) and mobile phase, comprising 30% acetonitrile (ACN), 70% water, and 0.25% trifluoroacetic acid (TFA), and compared to Copaxone release specifications.

#### 2.1.2. Molecular Weight Distribution (MWD)

MWD reflects the general distribution of the polypeptides in a complex mixture according to their size and relative abundance. The retention time of each component of the mixture on the size exclusion chromatographic (SEC) column depends on its hydrodynamic size, rather than the primary structure of the constituents. The Mylan FOGA samples were diluted to 4 mg/mL and evaluated using a Superose 12 column (GE Healthcare Bio-Sciences, Pittsburgh, PA, USA) with pH 1.5 (acidic) phosphate buffer mobile phase at 0.5 mL/min, with UV detection at 208 nm, and then compared to Copaxone release specifications.

#### 2.1.3. Coomassie Brilliant Blue (CBBG-250)

Differences in the mode of interaction between CBBG-250 dye molecules and glatiramer acetate (GA) peptides are indicative of different characteristic molecular charge distributions in polypeptide sequences. Mylan FOGA samples were mixed with a CBBG-250 dye solution at a 1:1 ratio and then centrifuged at 10,000 RCF (relative centrifugal force) for 30 min. The supernatant was diluted 6 to 100 with water, and absorbance was measured at 590 nm. Absorbance was calculated relative to a CBBG-250 dye control solution and compared to Copaxone release specifications.

### 2.2. High Resolution Methods

#### 2.2.1. RPLC-2D-MALLS

RPLC-2D-MALLS technique is utilized for the determination of the absolute molar mass of particles in solution by detecting the way in which they scatter light. To acquire a 2 dimensional (2D) analysis of the Copaxone mixture, the MALLS detector (MALL DAWN HELEO-II Wyatt Technology) was attached subsequent to the Reverse Phase High Performance Liquid Chromatography (RP-HPLC) system to provide elution profiles of molecular masses of Copaxone lots and Mylan FOGA lots as a function of hydrophobicity. Chromatographic separation of samples (concentration 10 mg/mL and injection volume 100 µL) was performed using a Purospher STAR RP-8e 5-µm 150 × 4.6 mm column (VWR), with gradient elution from 100% 0.1% TFA in water to 50% 0.1% TFA in acetonitrile over 60 min, at a flow rate of 1 mL/min. The elution profiles of the Mylan FOGA lots were compared to the range established for the sampling distribution of the Copaxone lots.

#### 2.2.2. Cation Exchange Chromatography (CEX)

CEX is based on a nondestructive separation of the polypeptide mixture according to the intensity of the positive charge of its components. The charged ions (or polar polypeptide molecules) are retained on the column based on their affinity to the negatively-charged stationary phase. For CEX, Mylan FOGA and Copaxone samples were diluted to 10 mg/mL, and 10-µL injections were run using a linear gradient from 50% 50 mM H_3_PO_4_ mobile phase (solution A) and 50% 50 mM Na_2_HPO_4_ (solution B) to 100% solution A over 20 min on a Propac WCX-10 5 μm 150 × 4.0 column (Thermo Scientific, Waltham, MA, USA) with detection at 210 nm.

#### 2.2.3. Atomic Force Microscopy (AFM)

AFM is a common technique to determine sample topography, such as aggregation forms [17]. The polypeptide mixtures were placed on negatively-charged plates and treated as previously detailed [12]. Images were analyzed and processed using Nanotec WSxM 5.0 (Nanotec Electronica S.L, Madrid, Spain).

#### 2.2.4. Viscotek TDAmax

Viscotek TDAmax, a multi-detector GPC/SEC system is used for a comprehensive conformational characterization of polymers and other macromolecules. Chromatographic separation of samples diluted to 5 mg/mL was performed at 30 ˚C using a Superose 12 column (GE Healthcare) with a pH 2.5 (acidic) phosphate buffer mobile phase at a 0.5 mL/min flow rate. The injection volume was 100 µL. The four parameters analyzed were: molecular weight and distribution (Mn, Mw, Mz), hydrodynamic radius (Rh; characterizes the average effective size of the peptides), intrinsic viscosity (IV; the contribution of the peptides to the overall viscosity of the tested solution, inverse of molecular density), and polydispersity (Pd; a measure of the uniformity of the polypeptide mixture with regard to the molecular weight distribution). OmniSEC software (version 5.1, Malvern Instruments Limited, Worcestershire, UK) was used for data processing.

#### 2.2.5. Size Exclusion Chromatography (SEC)

As a supportive cross test to the Viscotek assessments, polydispersity was measured using SEC. For this assessment, Mylan FOGA samples were diluted to 4 mg/mL and evaluated using a Superose 12 column (GE Healthcare) with pH 1.5 (acidic) phosphate buffer mobile phase at 0.5 mL/min, with UV detection at 208 nm (Copaxone release test conditions). The polydispersity was calculated and compared to the Copaxone 40 mg/mL lots.

### 2.3. Biological Characterization

#### 2.3.1. Potency ex vivo Cell-Based Assay

This assay reflects specific potency by measuring levels of a single cytokine (interleukin-2 (IL-2)) secreted by GA-primed T cells following the response to recall antigen (GA). Mice were immunized with Teva’s glatiramer acetate reference standard (GA RS); after 4 to 5 days, they were euthanized, and a primary culture of pooled spleen (SPL) cells was prepared. The SPL cells were activated in vitro with serial concentrations of either GA RS or a tested drug product lot (Mylan 40 mg/mL or Copaxone 40 mg/mL). After 24 h incubation, the cell supernatants were collected for Interleukin-2 (IL-2) measurement. The quantification of IL-2 secretion as a marker for cellular response was performed using a commercial Enzyme Linked Immunosorbent Assay (ELISA) kit. The reported result for each tested Mylan FOGA lot and Copaxone lot was calculated as relative to the reference standard (relative potency).

#### 2.3.2. In Vitro Cytotoxicity Assay using Human B Cell Lines

This assay determines the dose-dependent cytotoxic effect of tested products lots in serial concentrations by using an established human B cell-line. The cytotoxic effect of tested drug product lots (Mylan FOGA or Copaxone 40 mg/mL samples) on the Epstein-Barr virus (EBV)-transformed B-cell line was tested following two hours incubation with serial concentrations of either the GA RS or the tested drug product lot. Measurement of lactate dehydrogenase (LDH), a stable cytosolic enzyme that releases upon cell lysis was collected as a marker of cytotoxicity. The reported result for each tested lot was calculated as relative cytotoxicity values to the reference standard.

#### 2.3.3. Experimental Autoimmune Encephalomyelitis (EAE) Blocking Test

The EAE blocking test is used to evaluate the biological activity of glatiramer acetate lots by determining their ability to block the induction of EAE in mice. The encephalitic antigen used for EAE induction is the mouse spinal cord homogenate (MSCH). Blocking of EAE is defined as the reduction of the disease appearance (% Activity) and disease severity (mean maximal score ratio; MMS ratio).

#### 2.3.4. Anti-GA Antibody Recognition Assays

Two anti-GA antibodies bio-recognition assays based on Enzyme Linked Immunosorbent Assay (ELISA) for the specific bio-recognition of GA were employed using two anti-GA monoclonal antibodies (MAbs) in one assay and rabbit IgG polyclonal antibodies (PAbs) in the second assay. A microplate was coated with GA reference standard (GA RS) and tested drug product lot (Mylan FOGA or Copaxone). Following the coating and washing steps, the detection antibodies were added and incubated for 30 min at 37 °C. Then TMB was added and the optical density was measured. The results were expressed as the percent binding of the GA-specific monoclonal and polyclonal antibodies to drug product lots, relative to GA RS.

## 3. Results

### 3.1. Low Resolution Release Assays

The active ingredient in GA 40 mg/mL manufactured by Mylan/Natco was within the release specifications of the active ingredient in GA 40 mg/mL manufactured by Teva Pharmaceuticals Ltd for the three low resolution release measures employed; amino acid analysis, molecular weight distribution (except polydisperity) and Coomassie CBBG-250 (Table 1).

### 3.2. High Resolution PCC Methods

#### 3.2.1. RPLC 2D-MALLS

**Figure 1:**
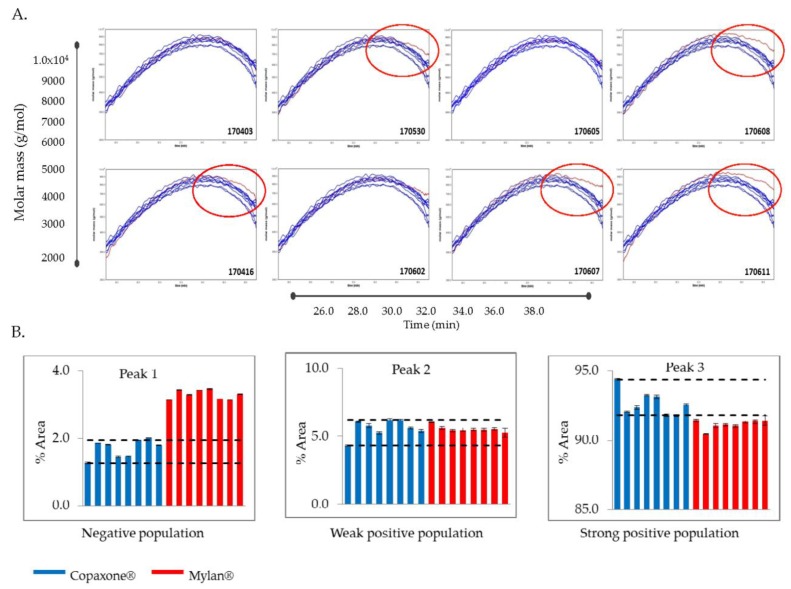
High Resolution physicochemical (PCC) methods (RPLC 2D-MALLS and CEX). Figure 1 illustrates the results of the high resolution PCC methods: (**A**) RPLC 2D-MALLS Elution profiles of molecular masses of Copaxone and Mylan 40 mg/mL lots as a function of hydrophobicity. Blue lines depict the eight tested Copaxone 40 mg/mL lots and the red line depicts the tested Mylan lot; the Mylan lot number is shown in each panel and red circles indicate Mylan lots outside the range of Copaxone lots; (**B**) CEX showed dissimilarities in surface charges for the Mylan lots. Blue bars represent the Copaxone 40 mg/mL lots and red bars represent the Mylan 40 mg/mL lots.

As shown in Figure 1A, the results of the Mylan lots fell into two groups, indicating compositional inconsistency among the lots. Five of the eight Mylan 40 mg/mL lots (170530, 170416, 170607, 170608, and 170611) were outside of the range established for Copaxone 40 mg/mL lots, and contained a high molecular weight hydrophobic population of peptides not found in Copaxone lots.

#### 3.2.2. Cation Exchange Chromatography

A typical Copaxone chromatogram includes three polypeptide subpopulations exhibiting strongly positive, weakly positive and negative overall charges [12]. In this experiment, the strongly positive-charged subpopulation for Copaxone comprises approximately 92% or more of the total mixture, while the negatively-charged and weak positive-charged subpopulations are present at smaller amounts. The polypeptide charge distribution in the Mylan 40 mg/mL lots were shown to be different from the Copaxone 40 mg/mL lots, as the strongly positively-charged subpopulation was consistently less than 92% of the total peptide content, and there was an increase in the amount of the negatively-charged subpopulation (Figure 1B). These findings are indicative of differences in overall polypeptide composition, including primary structure (amino acid sequence and its length).

#### 3.2.3. Atomic Force Microscopy

**Figure 2:**
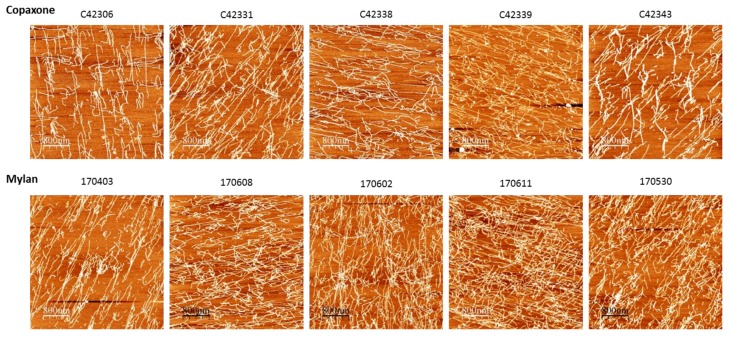
AFM. No differences seen in the aggregation patterns of different positively-charged polypeptide constituents adhered to a negatively-charged surface between Copaxone lots and Mylan/Natco 40 mg/mL lots.

AFM enables comparison between the aggregation patterns of different positively-charged polypeptide constituents adhered to a negatively-charged surface [17]. The morphology of the Mylan 40 mg/mL lots aggregates was similar to that of Copaxone, as shown with representative lots in Figure 2.

#### 3.2.4. Viscotek TDAmax

**Figure 3:**
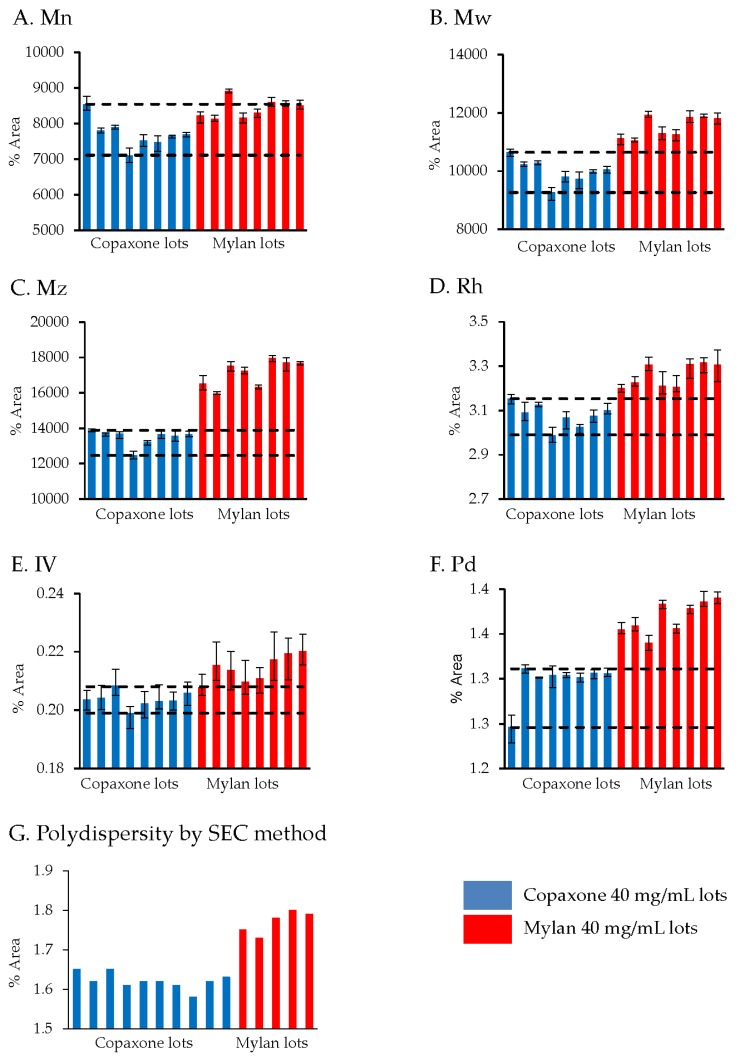
Analysis of molecular weight distribution and polydispersity between Mylan 40 mg/mL lots and Copaxone 40 mg/mL lots shown by Viscotek (**A**–**F**) and SEC (**G**).

Differences between Mylan 40 mg/mL lots and the Copaxone 40 mg/mL lots in molecular size, molecular weight and polydispersity were detected. As shown in Figure 3 Panels A–C, the molecular weight distribution parameters in Mylan are consistently higher, indicating a shift to higher molecular weight polypeptides. Likewise, the difference in polydispersity between Mylan and Copaxone was independently confirmed by an additional SEC measurement, where the mean value for the Mylan lots (1.77) was higher and outside of the characteristic Copaxone 40 mg/mL lots distribution parameters (mean 1.62, 75% upper quartile 1.54 and 25% lower quartile 1.490). This difference in polydisperity is indicative of the dissimilarity in the degree of the uniformity of polypeptides within the mixtures of the tested products (Figure 3F,G).

### 3.3. Biological Assays

#### 3.3.1. Potency Ex Vivo Cell Based Assay

**Figure 4:**
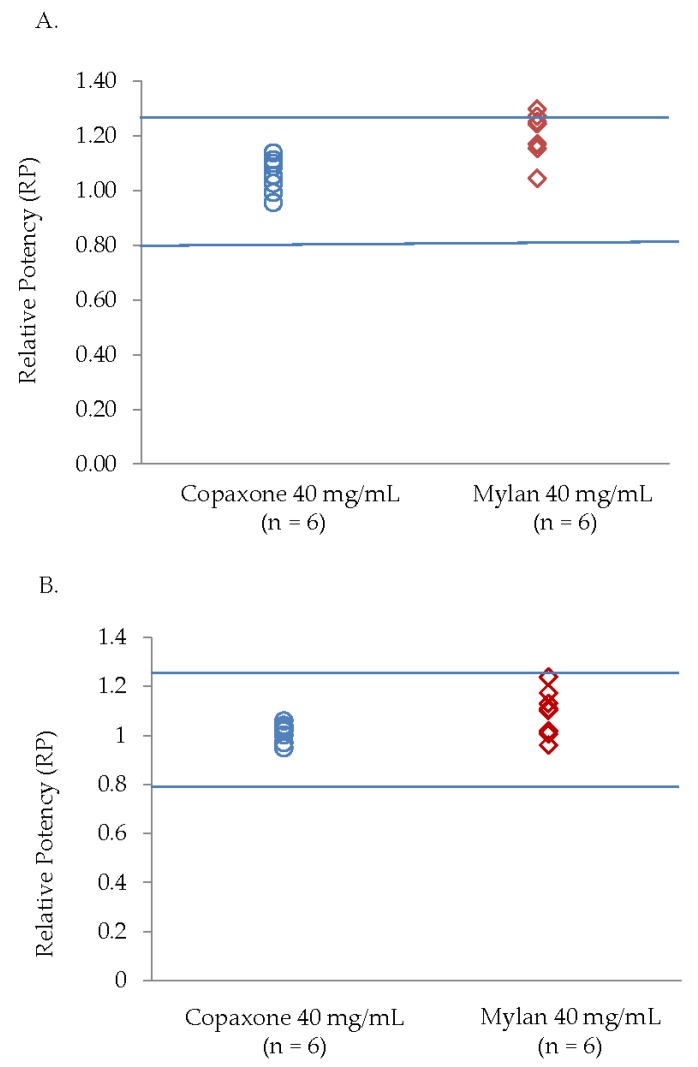
(**A**) Potency ex vivo cell-based assay and (**B**) Cytotoxicity cell-based activity assay.

Three Mylan 40 mg/mL lots (170608, 170611, 170530) were found to be above the Copaxone specification range for potency (Figure 4A). The RP for the Mylan lots were as follows: Lot 170602 1.243, Lot 170607 1.170, Lot 170608 1.271, Lot 170611 1.297, Lot 170530 1.252, Lot 170605 1.169, Lot 170403 1.044, and Lot 170416 1.153.

#### 3.3.2. Cytotoxicity Cell Based Assay

The Mylan 40 mg/mL lots were found to be within the Copaxone specifications for cytotoxicity, although there was a greater lot-to-lot variability (Figure 4B). The Mylan 40 mg/mL lots RP were as follows: Lot 170608 1.109, Lot 170611 1.174, Lot 170530 1.239, and Lot 170605 1.131.

#### 3.3.3. EAE Blocking Test

The results show that Mylan/Natco 40 mg/mL lots were within the Copaxone specifications for the EAE release method, with 100% activity and 0.0 MMS ratio for all lots. Noteworthy, the EAE assay cannot detect activity higher than 100% (i.e., 100% activity is the maximum).

#### 3.3.4. Anti-GA Antibodies Bio-Recognition Assays

The Mylan/Natco 40 mg/mL samples were within the Copaxone specifications for the mAbs-based release method, with percentage relative binding ranging from 98% to 107% for mAb 6B3/57 and from 97% to 108% for mAb 1C4/220, and for the polyclonal release method, with percentage relative binding ranging from 94% to 105%.

## 4. Discussion

In concordance with the filings provided by Mylan to the FDA [8], the active ingredient of the Mylan FOGA 40mg/mL lots were within the Copaxone specifications for some of the low resolution release tests such as the amino acid analysis, the molecular weight distribution and Coomassie CBBG-250. Additionally no differences were shown in the polypeptide aggregation patterns using AFM, the EAE blocking test, the cytotoxicity assay and in the antibody bio-recognition assays. However, comprehensive analyses of physicochemical and biological attributes orthogonally assessed by multiple independent and complementary methodologies indicate significant correlative differences between the active substances. These distinct compositional attributes, including in polydispersity and surface charge distribution and as well as the existence of high molecular weight hydrophobic polypeptides in Mylan FOGA 40 mg/mL lots, which were absent in Copaxone, were demonstrated using 2D-MALLS RP-HPLC, CEX, and Viscotek. In addition, biological differences were noted, and specifically a higher relative potency was found for three of the eight tested lots using an ex vivo potency assay.

In its recommendations for glatiramer acetate sameness evaluation published in “Draft Guidance for Glatiramer Acetate Injection”, the FDA referred to the molecular weight distribution parameters as one of the key physicochemical attributes needed to demonstrate API sameness [9]. The FDA draft guidance on glatiramer acetate sameness demonstration between the reference and the generic products includes an assessment of the molecular weight distribution parameters (the molar mass moments (Mn, Mw, Mz) and the polydispersity (Pd). While the general Mylan product molecular weight distribution profile was within the Copaxone specification range, using the non-specific low resolution SEC release method (except the polydispersity), significant differences in Mw and Mz were demonstrated using Viscotek, suggesting that the molecular weight distribution in the Mylan 40 mg/mL lots is shifted to the higher molecular weight peptides. Likewise there were differences in the polydispersity between the Mylan and Copaxone 40/mL mg lots as shown by SEC and by Viscotek, indicative of dissimilarities in the extent of the homogeneity of the polypeptide mixtures i.e., in the qualitative/quantitative composition of peptidic constituents. These observations were supported by the 2D-MALLS results which showed that five of eight Mylan 40 mg/mL lots had a high molecular weight hydrophobic population of peptides not found in the Copaxone 40 mg/mL lots.

The higher lot to lot variability of the Mylan 40 mg/mL lots in the 2D-MALLS analysis and the potency assay is also worth considering. Three of the five Mylan 40 mg/mL lots with the high molecular weight hydrophobic population of peptides also had relative potency values outside of the Copaxone specification limits in the ex vivo potency assay. Since these peptide subpopulations do not exist in representative Copaxone composition, their effect on clinical efficacy is not known, as there have been no clinical trials on the efficacy and safety of the Mylan FOGA 40 mg/mL product in comparison to Copaxone. CEX measures the surface charge of the polypeptides, one of the key attributes affecting the binding properties of antigens to their immunological counterpoints, i.e., antigen presenting cells and T cells [18]. Differences in the distributions of surface charge for a polypeptide between the Mylan FOGA 40 mg/mL and the Copaxone 40 mg/mL lots suggest that there are variations in the primary sequence or the overall mixture of the polypeptide, as the interaction of the polypeptides with the column depends on their overall surface charge interacting with the column. The fact that there are more negatively-surface-charged constituents in the active ingredient of the Mylan FOGA 40 mg/mL lots means that some of the polypeptides are arranged differently, and their spatial conformation caused shielding of some of the Lysines (positively-charged) constituents, such that the overall surface was less positively-charged relative to the active ingredient of Copaxone.

The CEX results for Mylan FOGA 40 mg/mL are in contrast to what we have previously reported for the active ingredient of the FOGA 20 mg/mL lots manufactured by Momenta (Sandoz, a division of Novartis) [12] and the FOGA product manufactured by Synthon [14].

The 20 mg/mL FOGA product manufactured by Momenta (Glatopa) and the Mylan FOGA 40 mg/mL examined in this report are both currently marketed as substitutable glatiramer acetate products in the US, and it is important to recognize these differences in polypeptide surface charge distribution among the substitutable generics, as well as relative to the branded glatiramer acetate product, Copaxone, and explore how these differences may influence the products’ immunogenicity properties. For instance, a study of pulmonary vaccines assessing polypeptide nanoparticles found that there were differences between the nanoparticles with positive and negative surface charges in their association with dendritic cells and their ability to promote a recruitment and maturation of lung dendritic cells [18]; and indeed, positively-charged particles have been shown to have a greater uptake by Jurkat cells, an in vitro model of immune T cells [19].

That the FOGA products manufactured by Momenta (Glatopa) and Mylan FOGA 40 mg/mL show differences in the physicochemical attributes relative to the active ingredient, GA in Copaxone and to each other, emphasizes that the manufacturing processes employed by different companies may lead to alterations in the primary structure of the active ingredient, which may not be revealed through the use of conventional methods and may require the use of the high resolution physicochemical measures described here. Indeed, we previously showed that FOGA products, Probioglat^® ^ (Probiomed, Mexico City Mexico, marketed in Mexico) and Polimunol^®^ (Synthon, marketed in Argentina) had alterations in some of these physicochemical attributes, as well as differences in immune response genes and pathways important for treating multiple sclerosis [11,15]. Subsequent to the publication of these findings, new guidelines were published in May 2016 by the Mexican Department of Health, requiring the use of the high resolution physicochemical and biological testing for the approval of GA generics [20]. The regulatory approval processes for FOGAs in Mexico differs from those in the US, countries in Europe and from Canada. The varied approach applied to the regulatory assessment of FoGAs in these different countries [21] underscores the challenges in determining a therapeutic equivalence for glatiramer acetate, a non-biological complex drug.

## 5. Conclusions

The use of multiple physicochemical and biological assays highlights important variances in the physical composition and biological functionality of the Mylan GA 40 mg lots and Copaxone GA 40 mg lots. The totality of evidence, including these data, demonstrates that the follow-on glatiramer acetate products, marketed to-date, consistently show some similarities in low-resolution tests, but the use of more sensitive higher-resolution methods and biological assays shows marked dissimilarities, and the effects of these alterations have not yet been assessed for their therapeutic implications for patients with multiple sclerosis.

## Figures and Tables

**Table 1:** Low resolution release test results for the Mylan 40 mg/mL lots.

**Amino Acid Analysis**					
**Parameter**	**170530**	**170602**	**170607**	**170608**	**170611**
l-Glu	0.141	0.140	0.140	0.142	0.142
l-Ala	0.433	0.437	0.435	0.436	0.433
l-Tyr	0.096	0.095	0.094	0.094	0.093
l-Lys	0.330	0.328	0.331	0.329	0.332
**Molecular weight distribution**					
**Parameter**	**170530**	**170602**	**170607**	**170608**	**170611**
RRT at -2SD	0.49	0.49	0.48	0.48	0.48
MW at -1SD	16100	15800	16800	17200	17200
MW at peak max	7450	7300	7900	8100	8100
**Coomassie Brilliant Blue (CBBG-250)**					
**Parameter**	**170530**	**170602**	**170607**	**170608**	**170611**
Relative Abs.	2.5	2.6	3.0	2.8	1.9
